# Enemy or friend: the personal and the factual patient-physician relationship

**DOI:** 10.3389/fmed.2023.1098305

**Published:** 2023-05-25

**Authors:** Frieder Keller, Ulla Ludwig, Markus Huber-Lang

**Affiliations:** ^1^Institute of Experimental and Clinical Pharmacology, Toxicology and Pharmacology of Natural Products, University Hospital, Ulm, Germany; ^2^Department of Internal Medicine, University Hospital, Ulm, Germany; ^3^Institute for Clinical and Experimental Trauma Immunology, University Hospital, Ulm, Germany

**Keywords:** patient-physician relationship, personal, anthropology, ethics, science

## Abstract

Physicians are expected to place the patient’s interests above their own. Such prioritization has worldwide consent. It constitutes the difference between medicine and other professions. The present conceptual opinion paper summarizes the authors’ clinical experience with patient care and student teaching during the last 45 years. The authors comment on their own conception by referring to present debates and prominent statements from the past. Fundamental changes in medicine have taken place over the last five decades. New diseases have emerged while diagnostic and therapeutic options for patients have grown steadily – along with healthcare costs. At the same time, economic and legal constraints for physicians have increased, as has moral pressure. The interaction of physicians with patients has gradually shifted from a personal to a factual relationship. In the factual, more formal relationship, the patient and physician represent equal partners of a legal contract, which jeopardizes the prioritization of the patient’s interests. The formal relationship implies defensiveness. By contrast, in the personal relationship, the physician adopts an existentialist commitment while simultaneously enabling and respecting the patient’s autonomous decision-making. The authors argue for the personal relationship. However, the patient and physician are no friends. Consequently, the physician in effect competes with the patient from a knowledge-based but opposite position. Both need to make efforts to consent and maintain the relationship even when they dissent. This implies that the physician does not simply comply with the patient’s wishes.

## 1. Introduction

The patient-physician relationship has fundamentally changed over the last 20 years. The time allocated to direct patient care decreased by one half, from 25 to 13% of physicians’ working hours (1). The trend toward digital and technical medicine clearly prevails (2). Even though the function of technology should ultimately be to foster the patient physician relationship (3); the associated factual thinking will result in a less personal and more formal relationship between legal partners of a contract (4). In the personal relationship, however, they are the subjects of an existential encounter. We propose tracing this relationship back to its anthropological roots.

## 2. Subsections relevant for the subject

Together, history-taking and the physical examination form the basis of a personal patient-physician relationship (5). Personal attention and narrative-based rather than inquisitorial history-taking already constitute therapy (6). In the course of such a narrative exploration, the patient better understands the interdependence of the facts by articulating them (7).

### 2.1. Empathy and emotions

In ancient Greece, the school of Knidos based medicine on facts, whereas the school of Kos based it on empathy (8). Empathy is difficult to teach and hard to learn, as measurements on the Jefferson scale have shown (9). In essence, empathy as an evolutionary advantage is inherited (10) – although it can be modulated by biographical and situational factors (11).

Modern brain imaging of mirror neurons and the theory of mind demonstrate how empathy can be visualized (12). Neural imaging has also revealed that some individuals behave altruistically more than others (13). Some express social competence; others appear callous (14). Alarmingly, altruism and aggression might represent only the extreme poles of a neuro-humoral continuum (15).

Empathy is personal at its core but must not be mistaken and equated with sympathy (11). With emotional distancing, the factual approach at first sight might outperform personal affection. However, the clarification of the conflictual nature of a personal patient-physician interaction will establish the required emotional distance.

### 2.2. Trust and responsibility

Emergency and technical medicine targets maximum quantity, often accepting average quality of results. Such factual medicine needs prioritization; and the conflict between utility and futility compels for debatable decisions (16). Codices regulate that physicians’ profiting from the patient’s emergency will undermine trust and is not compatible with physicians’ fiduciary responsibility in court (2).

The factual patient-physician relationship is necessarily based on confidence. Patients must trust in the physician’s competence and physicians need trust in their findings. Thus, invasive investigations must establish a reliable diagnosis. In order not to undermine patient’s confidence, advanced therapy might follow even without a reasonable prognosis or, worse, even where merely a palliative approach is indicated. Thus, factual medicine can result in overtreatment.

In contrast, the personal patient-physician relationship relies on responsibility (17). Responsibility is associated with disclosing the facts. Considering the patient’s whole history and all physical presentations produces insights. Insight requires less laboratory and less technical-diagnostic work; it makes untargeted and costly examinations superfluous to establish diagnosis and therapy. Thus, the personal relationship generates a resource-efficient method of diagnostics and treatment.

### 2.3. Patient’s primacy

The respect of autonomy is a modern achievement and this applies equally to patient and physician (2). According to the traditional and essential precondition of the patient-physician relationship, however, the interests of the patient should always come first, and the interests of the physician are legitimate but secondary (18). This assertion no longer applies in the formal patient-physician relationship, where both have equal rights jeopardizing the essential precondition of the patient’s primacy. In consequence, the currently spreading extreme of the formal model is commercialization (19).

The personal relationship is dyadic and discourse ethics constitute the procedural rules of respect, dialogue and consent (20). Through deliberative arguing, the shared decision-making process seeks an Habermasian paths of communicative action, whereas written informed consent asks only for the fact of an unrestricted yes or no. In the personal relationship, the physician does not simply deliver medicine – the physician embodies it. A sublime anthropological mechanism makes the physician seek a cure: Suffering or welfare on the patient’s part will correspondingly produce defeat or triumph on the physician’s part.

### 2.4. Concept and reason

The patient and physician are not friends (21). The patient-physician relationship is based not on amity but on a specific purpose, as already recognized by Plato (427–347 BC): This purpose is life and health, not the other person [Lysis 218d – 220b]. The physician must critically question what the patient presents to find the hidden secrets and see through, for instance, the mystery of non-adherence (22). The personal relationship makes the physician an enemy of the patient and the patient an enemy of the physician; patients intend to defend their integrity, but the physician’s actions are invasive.

By examining the individual history and touching the real body, physicians offer the personal relationship. Which relationship fits best is not up to the discretion of the physician alone and is not up to the discretion of the patient alone. To succeed, physicians must practice the culture of a controversial dispute in the personal relationship from the beginning.

### 2.5. Patient as foe, not friend

Disease is the adversary of both the patient and the physician. In fact, sick persons are enemies to themselves. The disease cannot be separated from the afflicted person; it cannot be attacked without attacking patients. By fighting the disease, the physician must combat the patient, who “does what the disease compels him to” (23). The more irritated by the course, the more patients will live in their own, disease-formed individual reality that encloses the patient like an “invisible cuticle” (24). Patients build subjective theories to protect their personal view on life and the world. Such theories represent parts of their mental survival strategy. What may objectively be judged a lie may subjectively sound to the patient like a plausible interpretation of what is happening to him or her.

Patients try to form a theory of the disease that relieves them not only of suffering but also of responsibility. Patients can repurpose illness to excuse and liberate themselves from external or internal obligations and pressing expectations. This social and mental relief imbues illness with a magical, seductive power. Patients try to locate the origin of a disease externally in the environment or to ascribe it to other persons. Typically, however, the exogenous alcohol does not cause it – the endogenous addiction causes the disease.

The disease is a product; it is created like an artwork. Disease is inevitably a part of the patient, and patients unconsciously identify themselves with their creations. The physician can heal the patient only through – metaphorically speaking – amputation of the disease. The patient-physician interaction will encounter interpersonal conflicts resulting from inherent contradictions of the patient’s own interests. These conflicts should be deconstructed by two sides: the patient and the physician. Familial and social bonds can contribute to inner conflict solving; often, however, they are also the reason of such conflicts (2).

### 2.6. Empowerment and competition

The personal relationship activates and empowers the patient to fight the disease. Empowerment of patients, however, results in the logic of competition (25). The patient and physician must fight rather than appease each other. Self-esteem prompts the patient to compete with the physician. This is also evident in the saying “medicine tastes bitter.” Similarly, medicine is not a game: it provokes aversion.

In a factual, formalism-based relationship, the physician aims to relieve symptoms and to comply with requests, as demonstrated by the US opioid crisis (26). In contrast, the personal relationship fights for a cure: The physician seeks to win, to defeat the disease and to heal. Thus, the physician must attack rather than appease.

The disease exposes the patient to the existential threat of annihilation, just as it exposes the physician to the existential risk of personal failure. When the patient is an adversary, the physician instinctively seeks a cure to render such personal risks unnecessary. When the patient is a customer within a value chain, the physician seeks a type of treatment that continues the clientship. This meets with the patient’s sublime resistance to any expropriation of his/her discharge-like retooling of the disease.

Patients might feel ambivalent about a medical cure that liberates them from threats but re-exposes them to interpersonal and existential demands. The personal relationship also exposes the physician to deep irritations, even harboring potential risks to the physician’s self-confidence and self-esteem. The risk of interpersonal war’s absurdities (19) must be prevented by flexibly shifting from a personal to the strictly factual mode of interaction.

## 3. Discussion

Martin Buber (1878–1965) argued for an existentialist anthropology but stated that there exists a normative limitation of mutuality in medicine. Accordingly, fundamental, not just practical, facts separate the physician’s and the patient’s person (27). As Axel Honneth (* 1949) analyzed the grammar of social conflicts, also the patient-physician relationship is a “struggle for personal recognition, appreciation and acknowledgment” [SfR. 1992: 264]. However, in the fight with the patient, the weapon is science, not animosity.

### 3.1. Science of the individual

The personal patient-physician relationship considers each patient as a new and special case. In the factual relationship, however, the patient is a representative of general knowledge. Rational thinking underlies both the technical and personal approach to patient care, but the logic differs between symptom-oriented and patient-oriented approaches. Whereas the factual relationship applies technology, the personal relationship applies science. Guidelines and standard operating procedures might protect against neglect, but they can distract from personal engagement.

The personal relationship requires a clinical scientist to individualize medicine. The personal encounter follow a cognitive timeline spanning past, present and future. The clinical practice resembles an epistemological process like a doctoral thesis. According to Michel Foucault (1926–1984), it was not until the 19th century, “that such medicine was borne that is science of the individuality” [BoC. 1973. Epilogue]. Diseases are rule-based entities – otherwise, medicine as a science would be impossible. The disease identified does not differ from textbook knowledge, but patients make them appear real in their own different and individual way.

As medical scientists, physicians need to rediscover and reinvent how they can apply general knowledge to the individual case. Precision medicine looks at the facts, while individualized medicine looks at the person. The personal approach considers all findings of the individual, not just precise and measurable findings.

Physicians make mistakes. In this context, Michel Foucault stated, “physicians learn more by their failures than from their success” [BoC. 1973, IV; 5]. Consequently, in a factual relationship, any fault must be adequately compensated, whereas in a personal relationship, failure must be communicated. Again, patients and physicians are not friends. Compensation, however, means money, whereas communication seeks a medical solution.

### 3.2. Ethics and law

**Figure 1:**
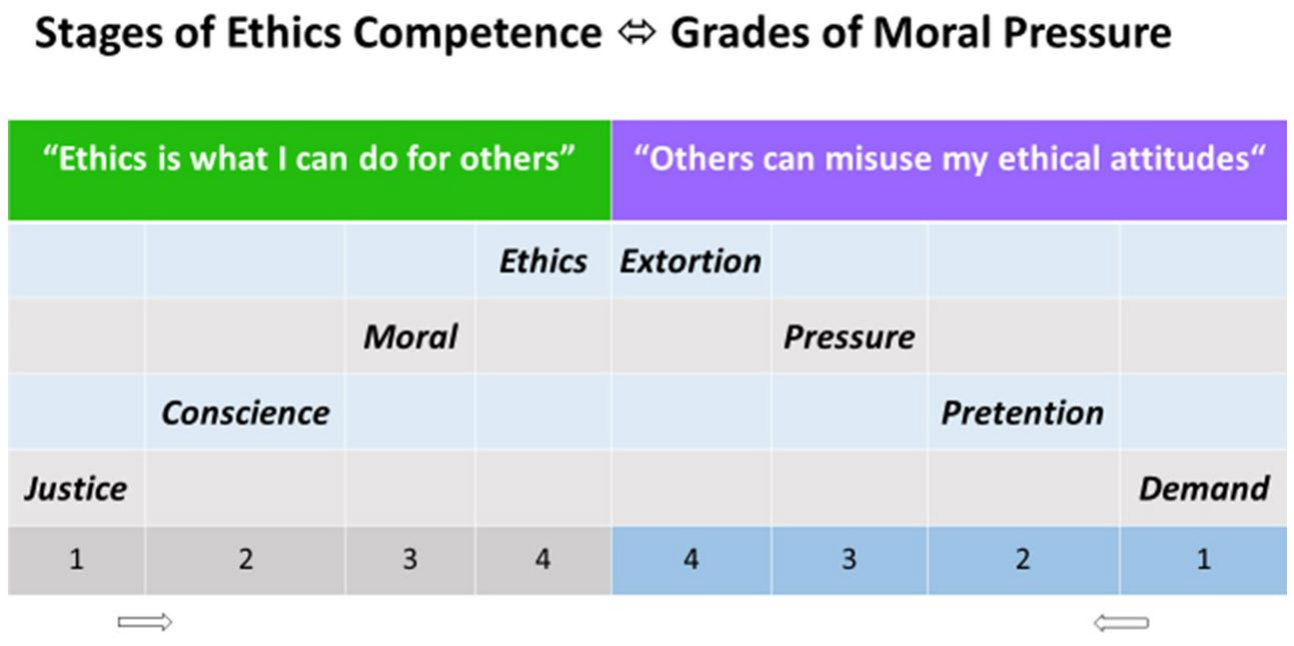
The 4 stages of competence in ethics analogous to the theory of moral development proposed by Lawrence Kohlberg (1927–1987). Complementary, also 4 grades of moral pressure can be identified.

Ethical competence stands above moral obligations, as discussed with respect to the dilemmas of truth telling, abortion, or terminal sedation and brain death (17). The personal relationship, more than the factual relationship, therefore, is associated with ethical stress (16). Patients can exert moral pressure thus potentially exploiting the physician’s ethical attitudes (Figure 1).

Physicians will deliver high-stage ethics and grand epikeia, a place above the law, only in singular cases. Supererogation can be expected at the personal, not at the formal level. Conversely, the higher the moral pressure exerted by the patient, the lower the stage of ethical response by the physician, ultimately becoming defensive, purely legal and factual. Moral pressure and the potential for exploitation indicate yet again that the patient is not the friend of the physician: “the patient is the enemy” a nephrology fellow once stated. However, the same ethical principles apply to an enemy as to a friend.

### 3.3. Subjective reality and objective truth

Patients may feel comforted when physicians act like a substitute for their mother or father. Such a feeling constitutes an anthropologic motion for the personal relationship with physicians. The more dramatically the conditions deteriorate, the more urgently physicians are expected to represent an almighty savior granting eternal life.

Unaffected by familial expectations and transcendental projections physicians must confront the individual reality of the patient with the objective reality. Carl Friedrich von Weizsäcker (1912–2007) reminded the lay public that the “biological purpose of a disease is ultimately to make the individual organism die” [AoP. 1977. I, 9.]. In contrast to the biological purpose (=causa finalis), the biographical meaning of sickness is an unpleasant appeal (=causa efficiens): The patient should change something, either lifestyle, workplace, relations, or attitudes.

Modern medicine opens a global perspective of unprecedented possibilities. For an individual, however, the future prospect narrows by each day that passes. A factual relationship causes the discrepancy between common sunrise and private sunset to be forgotten – not so the personal.

## 4. Conclusion

The personal, patient-centered relationship will have diagnostic, therapeutic and prognostic advantages over the factual relationship (28). The personal relationship is like chess, but serious rather than a game; it is like war, but healing rather than killing the means; the patient is an adversary rather than an accomplice, and the physician is a combatant not a comrade. However, unlike all other human relationships, this existential confrontation will result in either success or failure for both sides simultaneously.

## Author contributions

FK had the primary idea on friend or foe. UL strengthened the participatory approach. MH-L contributed historical and economical aspects and focused on traditional and triage ethics. All authors contributed to the article and approved the submitted version.

## Funding

The open access publication cost will be covered by Ulm University.

## Conflict of interest

The authors declare that the research was conducted in the absence of any commercial or financial relationships that could be construed as a potential conflict of interest.

## Publisher’s note

All claims expressed in this article are solely those of the authors and do not necessarily represent those of their affiliated organizations, or those of the publisher, the editors and the reviewers. Any product that may be evaluated in this article, or claim that may be made by its manufacturer, is not guaranteed or endorsed by the publisher.

## Abbreviations

AoP: Ambivalence of Progress
BC: before Christ
BoC: The Birth of the Clinic
SfR: Struggle for Recognition

## References

[1] Chaiyachati KH Shea JA Asch DA Liu M Bellini LM Dine CJ. Assessment of inpatient time allocation among first-year internal medicine residents using time-motion observations. JAMA Intern Med. (2019) 179:760–7. doi: 10.1001/jamainternmed.2019.0095, PMID: 30985861 PMC8462976

[2] Derse AR. The physician-patient relationship. N Engl J Med. (2022) 387:669–72. doi: 10.1056/NEJMp2201630 35984346

[3] Simini F. Medicine based engineering and informatics to Foster patient physician relationship In: Simini F Bertemes-Filho P, editors. Medicine-based informatics and engineering, Lecture Notes in Bioengineering. Cham: Springer (2022)

[4] Berger R Bulmash B Drori N Ben-Assuli O Herstein R. The patient-physician relationship: an account of the physician's perspective. Isr J Health Policy Res. (2020) 9:33. doi: 10.1186/s13584-020-00375-4, PMID: 32605635 PMC7325021

[5] Lown B. The Lost Art of Healing: Practicing Compassion in Medicine. New York, NY: Ballantine Books (1999).

[6] Moore W. Applying applied ethics through ethics consulting. Transfus Apher Sci. (2010) 42:209–14. doi: 10.1016/j.transci.2010.01.020 20227344

[7] Hafen BQ Karren KJ Frandsen KJ Smith NL. Mind/Body Health: The Effects of Attitudes, Emotions, and Relationships. Boston: Allyn and Bacon (1996).

[8] Turgut M. Ancient medical schools in Knidos and Kos. Childs Nerv Syst. (2011) 27:197–200. doi: 10.1007/s00381-010-1271-2 20737273

[9] Alhassan M. Effect of a 2-day communication skills training on nursing and midwifery students' empathy: a randomized controlled trial. BMJ Open. (2019) 9:e023666. doi: 10.1136/bmjopen-2018-023666, PMID: 30826757 PMC6429730

[10] Braadbaart L de Grauw H Perrett DI Waiter GD Williams JH. The shared neural basis of empathy and facial imitation accuracy. NeuroImage. (2014) 84:367–75. doi: 10.1016/j.neuroimage.2013.08.061 24012546

[11] Neumann M Bensing J Mercer S Ernstmann N Ommen O Pfaff H. Analyzing the "nature" and "specific effectiveness" of clinical empathy: a theoretical overview and contribution towards a theory-based research agenda. Patient Educ Couns. (2009) 74:339–46. doi: 10.1016/j.pec.2008.11.013 19124216

[12] Jeon H Lee SH. From neurons to social beings: short review of the Mirror neuron system research and its socio-psychological and psychiatric implications. Clin Psychopharmacol Neurosci. (2018) 16:18–31. doi: 10.9758/cpn.2018.16.1.18, PMID: 29397663 PMC5810456

[13] Marsh AA Stoycos SA Brethel-Haurwitz KM Robinson P VanMeter JW Cardinale EM. Neural and cognitive characteristics of extraordinary altruists. Proc Natl Acad Sci U S A. (2014) 111:15036–41. doi: 10.1073/pnas.1408440111, PMID: 25225374 PMC4210306

[14] Brethel-Haurwitz KM Cardinale EM Vekaria KM Robertson EL Walitt B VanMeter JW. Extraordinary altruists exhibit enhanced self-other overlap in neural responses to distress. Psychol Sci. (2018) 29:1631–41. doi: 10.1177/0956797618779590, PMID: 30130165 PMC6180668

[15] Marsh AA. The caring continuum: evolved hormonal and proximal mechanisms explain prosocial and antisocial extremes. Annu Rev Psychol. (2019) 70:347–71. doi: 10.1146/annurev-psych-010418-103010, PMID: 30231001

[16] Martin DE Parsons JA Caskey FJ Harris DCH Jha V. Ethics of kidney care in the era of COVID-19. Kidney Int. (2020) 98:1424–33. doi: 10.1016/j.kint.2020.09.014, PMID: 33038425 PMC7539938

[17] Stahl RY Emanuel EJ. Physicians, not conscripts-conscientious objection in health care. N Engl J Med. (2017) 376:1380–5. doi: 10.1056/NEJMsb1612472, PMID: 28379789

[18] Thompson DF. Understanding financial conflicts of interest. N Engl J Med. (1993) 329:573–6. doi: 10.1056/NEJM199308193290812 8336759

[19] Shutzberg M. The doctor as parent, partner, provider … or comrade? Distribution of power in past and present models of the doctor-patient relationship. Health Care Anal. (2021) 29:231–48. doi: 10.1007/s10728-021-00432-2, PMID: 33905025 PMC8322008

[20] Keller F Allert G Baitsch H Sponholz G. Ethics in medicine working Group at the University of Ulm. Discourse ethics in practical medicine. Med Humanit. (2006) 32:99–103. doi: 10.1136/jmh.2005.000224 23673802

[21] Temperance KK Friendship M Cessation S. Temperance, moral Friendship, and smoking Cessation. J Med Philos. (2019) 44:299–313. doi: 10.1093/jmp/jhz003 31102454

[22] Mac Laughlin EJ Raehl CL Treadway AK Sterling TL Zoller DP Bond CA. Assessing medication adherence in the elderly: which tools to use in clinical practice? Drugs Aging. (2005) 22:231–55. doi: 10.2165/00002512-200522030-00005 15813656

[23] Galen Commentary on Hippocrates’ Epidemics Book I part I-III. Edition of the Arabic Version with English Translations and Notes by Uwe Vagelpohl. De Gruyter, Berlin (2014): 271–273.

[24] von Uexküll T. Anthropology and the theory of medicine. Theor Med. (1995) 16:93–114. doi: 10.1007/BF00993789 7652714

[25] Audrain-Pontevia AF Menvielle L. Do online health communities enhance patient-physician relationship? An assessment of the impact of social support and patient empowerment. Health Serv Manag Res. (2018) 31:154–62. doi: 10.1177/0951484817748462 29280679

[26] Stratton TP Palombi L Blue H Schneiderhan ME. Ethical dimensions of the prescription opioid abuse crisis. Am J Health Syst Pharm. (2018) 75:1145–50. doi: 10.2146/ajhp170704, PMID: 30045851

[27] Scott JG Scott RG Miller WL Stange KC Crabtree BF. Healing relationships and the existential philosophy of Martin Buber. Philos Ethics Humanit Med. (2009) 4:11. doi: 10.1186/1747-5341-4-11, PMID: 19678950 PMC2733137

[28] Little P Everitt H Williamson I Warner G Moore M Gould C. Observational study of effect of patient centredness and positive approach on outcomes of general practice consultations. BMJ. (2001) 323:908–11. doi: 10.1136/bmj.323.7318.908, PMID: 11668137 PMC58543

